# Seasonal effect on the incidence of post-operative wound complications after trauma-related surgery of the foot, ankle and lower leg

**DOI:** 10.1007/s00402-020-03395-6

**Published:** 2020-03-09

**Authors:** Fay Ruth Katharina Sanders, Mirjam van’t Hul, Rosanne Maria Güzelleke Kistemaker, Tim Schepers

**Affiliations:** Amsterdam UMC, University of Amsterdam, Trauma Unit, Meibergdreef 9, 1105 AZ Amsterdam, The Netherlands

**Keywords:** Seasonality, Wound complication, Surgical site infection, Fracture related infection, Foot, Ankle, Trauma surgery

## Abstract

**Introduction:**

Post-operative wound complications remain among the most common complications of orthopedic (trauma) surgery. Recently, studies have suggested environmental factors such as season to be of influence on wound complications. Patients operated in summer are reported to have more wound complications, compared to other seasons. The aim of this study was to identify if “seasonality” was a significant predictor for wound complications in this cohort of trauma-related foot/ankle procedures.

**Materials and methods:**

This retrospective cohort study included all patients undergoing trauma-related surgery (e.g. fracture fixation, arthrodesis, implant removal) of the foot, ankle or lower leg. Procedures were performed at a Level 1 Trauma Center between September 2015 until March 2019. Potential risk factors/confounders were identified using univariate analysis. Procedures were divided into two groups: (1) performed in summer (June, July or August), (2) other seasons (September–May). The number of surgical wound complications (FRIs, SSIs or wound dehiscence) was compared between the two groups, corrected for confounders, using multivariate regression.

**Results:**

A total of 599 procedures were included, mostly performed in the hindfoot (47.6%). Patients were on average 46 years old, and mostly male (60.8%). The total number of wound complications was 43 (7.2%). Age, alcohol abuse, open fracture and no tourniquet use were independent predicting factors. No difference in wound complications was found between summer and other seasons, neither in univariate analysis [4 (3.2%) vs 39 (8.2%), *p* = 0.086] nor when corrected for predicting factors as confounders (*p* = 0.096).

**Conclusions:**

No seasonality could be identified in the rate of wound complications after trauma surgery of the lower leg, ankle and foot in this cohort. This lack of effect might result from the temperate climate of this cohort. Larger temperature and precipitation differences may influence wound complications to a larger extent. However, previous studies suggesting seasonality in wound complications might also be based on coincidence.

## Introduction

The incidence of surgical site infections (SSIs) following orthopedic trauma surgery of foot and ankle has been reported as ranging from 0 to 9.4% [1]. In complex foot injuries, this percentage can even increase up to 25% [2]. This incidence is much higher compared to most other surgical procedures and is most likely related to the thin soft-tissue envelope and damage to vascularization during the injury [3]**.** Moreover, the fact that a fracture is involved, could have implications for the odds of developing surgical wound complications or, in recently defined terminology: “fracture related infection (FRI) [4]. Surgical wound complications, including FRIs and other SSIs can cause longer hospital stay or readmission, increased use of antibiotics and revision surgery, resulting in higher healthcare costs [5]. With the increasing rate of antibiotic resistance, preventing these complications is becoming even more important.

Various risk factors for developing surgical wound complications have been described. Risk factors can be divided in patient-related factors, injury-related factors, procedure-related factors and other risk factors [6]. The last group contains environmental factors such as geographical region, socioeconomic status of the country and season [7, 8].

A seasonal effect on the incidence of infectious complications after different types of surgery has been increasingly described in recent literature [8–18]. Numerous publications have documented a significantly higher incidence of wound complications during the summer months [10, 16, 19–21]. The suggestion is that in summer, the warmer temperatures and higher humidity of the air, provide optimal conditions for proliferation of bacteria outside of the operation room. Moreover, when the surface of the skin is moist and warm, bacterial growth is stimulated [22]. Considering the development of global warming, we also might have to anticipate on a growing number of wound complications. If this seasonal effect is indeed present for all types of surgery, it could be used to lower the overall rate of wound complications by for example planning of elective procedures.

The purpose of this study was to identify if there is a seasonal difference in the number of surgical wound complications after orthopedic trauma-related foot/ankle surgery. The hypothesis was that there are significantly more wound infections during the summer months.

## Materials and methods

In this retrospective cohort study, all patients undergoing trauma-related surgery of the foot, ankle or lower leg, were included. Data were anonymously collected from electronic patient records operated between September 2015 and March 2019 at a single level-1 trauma center in the Netherlands. The surgical procedures performed included open fracture reduction and fixation (ORIF), primary and secondary arthrodesis, repairing of acute tendon ruptures, and implant removal. Both acute and elective surgeries were included, as long as the initial diagnosis was trauma-related. Follow-up after surgery had to be at least 90 days for a patient to be eligible.

Permission for the use of data was acquired from all eligible patients. The study protocol was checked by the hospital’s privacy advisor and the study was registered at the Central Register for Data Processing. Exclusion criteria were: age below 18 years old, preexistent wound complication before surgery, and one of the following surgical procedures: external fixation, percutaneous wire fixation only, decompression of acute compartment syndrome, and treatment of Charcot arthropathy. In addition, non-trauma related procedures like amputations (toe, foot or lower leg), exostosis operations and arthroscopy were also excluded.

### Methods

Baseline patient, injury and surgical characteristics were collected based on procedure. If one patient had multiple surgical procedures in the study period, these were counted separately. Bilateral surgery was also counted as two separate procedures since there would be two incisions with the potential to infect. The following patient’s baseline characteristics were collected: gender, age, height, weight, American Society of Anesthesiologists (ASA) classification, immune disorders or use of immunosuppressive medication, severe comorbidities (including Diabetes Mellitus, severe kidney failure defined as eGFR < 20), and intoxications (smoking, alcohol and drugs abuse).

The injury characteristics collected for fractures were: open/closed fracture, Gustilo classification and type of fracture, and for all injuries the inflicted body part:

- *Forefoot* Metatarsal fracture.
- *Midfoot* Navicular fracture, Cuboid fracture, Lisfranc and Chopart dislocation/fracture.
- *Hindfoot* calcaneus fracture, talus fracture.
- *Lower leg* Tibia plateau fracture, tibia fracture, Cruris fracture, fibula fracture, tendon injury.
- *Ankle* Pilon fracture, Maisonneuve fracture, Weber A/B/C fracture, tendon injury.

Surgical characteristics were: previous procedure in same area, type of procedure (ORIF, arthrodesis, implant removal, other), the amount of days between trauma and procedure (for acute procedures only), duration of surgery in minutes, tourniquet use, the use/dose of prophylactic antibiotics, type of fixation (plates, nails and/or screws), use of bone void fillers, and amount of blood loss in ml. Post-surgical characteristics included the use of antibiotics, type of wound dressing (plaster cast, pressure bandage, vacuum system) and time to mobilization.

All procedures were categorized in the month of surgery and then coded as summer (June–July–August) or other seasons (September–May). Weather conditions for that time of year were obtained from the Dutch Weather Institute or “Koninklijk Nederlands Meteorologisch Instituut” (KNMI). Conditions entailed average temperatures, precipitation and hours of sun.

### Outcome

The primary outcome was the incidence of wound complications, defined as a SSI according to US Centers for Disease Control and Prevention (CDC) criteria [23] or another surgical wound healing complication (wound dehiscence), developed within 90 days after the surgical procedure. The term FRI was not used as a primary endpoint since this study also includes data from secondary/elective procedures (sometimes years after the initial fracture occurred) and the definition of FRI does not clearly describe a time frame for the diagnosis. Wound complications were diagnosed during follow-up visits, protocolled at 2 weeks, 6 weeks and 3 months after discharge from the hospital and retrospectively retrieved from the medical records. All patients were instructed upon discharge to contact the hospital sooner in case of suspicion of wound complications. In case of a complication, treatment was recorded as well, categorized as expectative treatment (wait and see), vacuum system, wound reopened (at outpatient clinic or ward), oral antibiotics, i.v. antibiotics or revision surgery.

### Statistical analysis

Data were collected and analyzed using SPSS, Version 25.0 [24]. All clinically relevant patient, injury and (post-)surgical variables were univariately analyzed for differences between patients with and without wound complications to identify possible confounders. Chi-square analysis was used to identify differences for categorical variables. For continuous data that were normally distributed a student’s *T*-test was used, and when not normally distributed, a Mann Whitney *U* test. All (nearly) significantly differing variables (*p* < 0.2) were included in multivariate analysis to identify individual predictors for wound complications with manual backwards selection. To limit bias caused by missing data, multiple imputations was used for variables with (under 50%) missing data. Imputation was performed using predictive mean matching with 10 sets. Imputed variables were: height, weight, smoking, alcohol abuse, drugs abuse, ASA classification, previous surgery, prophylactic antibiotics, duration of surgery, tourniquet use, primary closure, wound dressing and days between injury and surgery (for acute procedures). The amount of blood loss was not imputed because of too many missing values. After imputation, the remaining individual predictors were added to multivariable logistic regression analyzing the effect of season of the number of wound complications. The pooled results of the ten imputed sets were used to estimate odds ratios and confidence intervals.

Statistical significance was defined as *p* < 0.05. Due to practical considerations regarding the implementation of the electronic medical record system in 2015, all data was collected from that point onwards and no sample size calculation was performed before the start of the study.

## Results

**Fig. 1:**
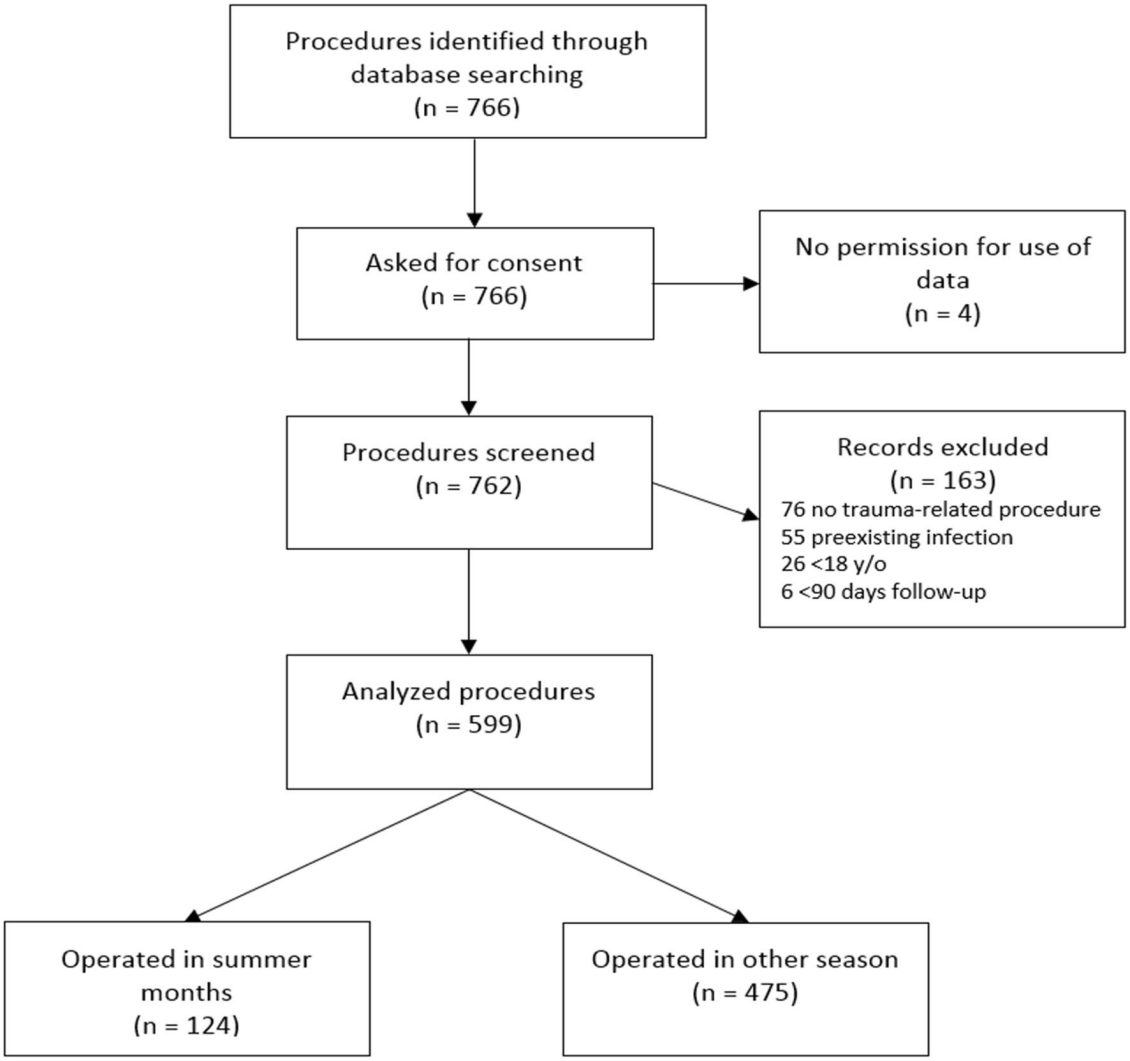
Flow diagram of patient selection

A total of 766 surgical procedures were reviewed for this study. Based on the exclusion criteria and the possibility for the patient to object, a final 599 procedures in 498 patients were included for analysis (Fig. 1). In this cohort, the majority was male (60.8%), the mean age was 46 (IQR 31–57) and most procedures were located in the hindfoot (45.1%) followed by the ankle (22.7%). Some 43 patients (7.2%) developed a surgical wound complication, of which 22 were deep infections, 10 superficial infections and 11 wound dehiscence. Excluded cases did not differ in gender or age but had Diabetes Mellitus more often and were less likely to be smoking tobacco or using drugs (Table 5 of “Appendix”).

Of all patients (*n* = 124) operated during the summer months, 4 (3.2%) developed a wound complication. In the other months (*n* = 475) the number of wound complications was 49 (8.2%). Table 1 shows wound complications for each season individually. Tables 2 and 3 contain patient, injury and surgical characteristics of all included patients. When comparing these characteristics between cases resulting in a wound complication and cases who did not, age, ASA classification, alcohol abuse, the number of open fractures, and tourniquet use differed significantly. In addition, gender, duration of surgery and amount of blood loss were not significantly differing but had a p value below 0.2, therefore, also qualifying for inclusion in multivariate analysis. Because the amount of blood loss was missing in over 50% of procedures (317), this variable was not imputed or included in multivariate analysis. After backwards selection, the variables age, alcohol abuse, open fracture and tourniquet remained as independent predictors of wound complications. As shown in Table 4, correcting for these variables as confounders, there was no statistically significant relation between season of surgery and wound complications (*p* = 0.096).

**Table 1:** Number of wound complications per season

	Winter (*n* = 186)	Spring (*n* = 123)	Summer (*n* = 124)	Fall (*n* = 166)
Complications	17 (9.2%)	9 (7.3%)	4 (3.2%)	13 (7.8%)
Deep SSI	8 (4.3%)	7 (5.7%)	4 (3.2%)	3 (1.8%)
Superficial SSI	5 (2.7%)	1 (0.8%)	0	4 (2.4%)
Dehiscence	4 (2.2%)	1 (0.8%)	0	6 (3.6%)

*SSI* surgical site

**Table 2:** Patient characteristics

	No wound complication (*n* = 556)	Wound complication (*n* = 43)	Significance
Gender, no. (%) male	343 (61.7%)	21 (48.8%)	0.133
Age, median [IQR]	45 [31–57]	54 [39–61]	0.008*
BMI, median [IQR]^a^	24.8 [22.7–28.1]	25.5 [23.3–29.2]	0.433
Diabetes, no. (%)	16 (2.9%)	1 (2.3%)	1.000
Immunocompromised, no. (%)	11 (2.0%)	2 (4.7%)	0.538
ASA-classification^b^			0.008*
I–II	522 (94.9%)	36 (83.7%)	
III–IV	28 (5.1%)	7 (16.3%)	
Smoking, no. (%) of smokers^c^	170 (38.0%)	16 (50.0%)	0.248
Alcohol abuse (> 2 units/day), no. (%)^d^	36 (9.0%)	7 (22.6%)	0.034*
Drug use, no. (%)^e^	40 (10.1%)	3 (9.7%)	0.1000

*IQR* inter quartile range, *n* number of included procedures, *No* number, *%* percentage of, > more than

*Statistically significant difference (*p* < 0.05)

^a^Missing 124 (114 in no wound complication, 10 in wound complication)

^b^Missing 6 (in no wound complication)

^c^Missing 120 (109 in no wound complication, 11 in wound complication)

^d^Missing 168 (156 in no wound complication, 12 in wound complication)

^e^Missing 170 (158 in no wound complication, 12 in wound complication)

**Table 3:** Injury and surgical characteristics

	No wound complication (*n* = 556)	Wound complication (*n* = 43)	Significance
Open fracture, no. (%)	49 (8.8%)	9 (20.9%)	0.020*
Part of leg^a^			
Forefoot	23 (4.1%)	1 (2.3%)	0.691
Midfoot	77 (13.8%)	4 (9.3%)	
Hindfoot	250 (45.0%)	20 (46.5%)	
Ankle	127 (22.8%)	9 (20.9%)	
Lower leg	79 (14.2%)	9 (20.9%)	
Previous surgery, no. (%)^b^	124 (23.1%)	9 (22.0%)	1.000
Acute operation			
Acute	347 (62.4%)	28 (65.1%)	0.849
Elective	209 (37.6%)	15 (34.9%)	
Type of surgery			
Osteosynthesis	343 (61.7%)	25 (58.1%)	0.694
Arthrodesis	91 (16.4%)	7 (16.3%)	
Implant removal	94 (16.9%)	7 (16.3%)	
Other procedures	28 (5.0%)	4 (9.3%)	
Days between injury and operation, median [IQR]^c^	7 [3–14]	7 [1–16]	0.732
Duration of surgery, median [IQR]^d^	75 [55–110]	89 [60–138]	0.101
Tourniquet use, no. (%)^e^	228 (41.1%)	7 (16.3%)	0.002*
Prophylactic antibiotics^f^			
Yes	456 (82.9%)	34 (79.1%)	0.203
No	92 (16.7%)	8 (18.6%)	
Therapeutic	2 (0.4%)	1 (2.3%)	
Blood loss in mL, Median [IQR]^g^	100 [50–200]	200 [75–375]	0.036*
Primary closure^e^	550 (99.1%)	43 (100%)	1.000
Post-surgical dressing^h^			0.525
Pressure bandage	344 (63.8%)	26 (61.9%)	
Cast	146 (27.1%)	10 (23.8%)	
Negative pressure system	49 (9.1%)	6 (14.3%)	

*IQR* inter quartile range, *n* number of included procedures, *No* number, *%* percentage of

*Statistically significant difference (*p* < 0.05)

^a^Missing: 1 (in no wound complication, crush injury)

^b^Missing 21 (19 in no wound complication, 2 in wound complication)

^c^Only for acute injuries, missing: 9 (5 in no wound complications, 4 in wound complication), *N* = 366

^d^Missing 57 (56 in no wound complication, 1 in wound complication)

^e^Missing 1 (in no wound complication)

^f^Missing 6 (in no wound complication)

^g^Missing 317 (291 in no wound complication, 26 in wound complication)

^h^Missing 18 (17 in no wound complication, 1 in wound complication)

**Table 4:** Multivariate regression

	Odds ratio	95% CI	*p*-value
Age	1.029	1.007–1.051	0.010
Alcohol abuse	2.680	1.065–6.747	0.037
Open fracture	2.559	1.109–5.904	0.028
Tourniquet	0.297	0.128–0.690	0.005
Summer season	2.485	0.851–7.258	0.096

*CI* confidence interval, *%* percentage of

**Fig. 2:**
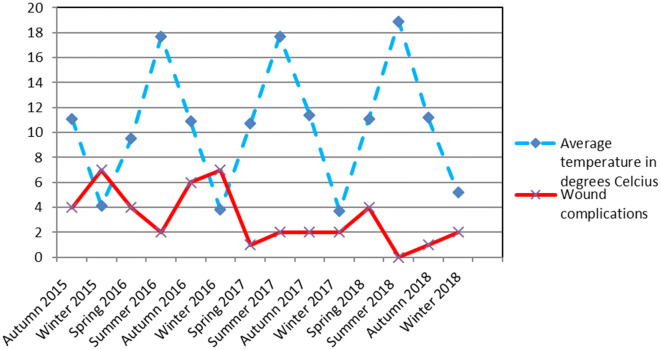
Average temperatures in degrees Celcius and amount of wound complications during the study period *Source* Koninklijk Nederlands Meteorologisch Instituut

**Fig. 3:**
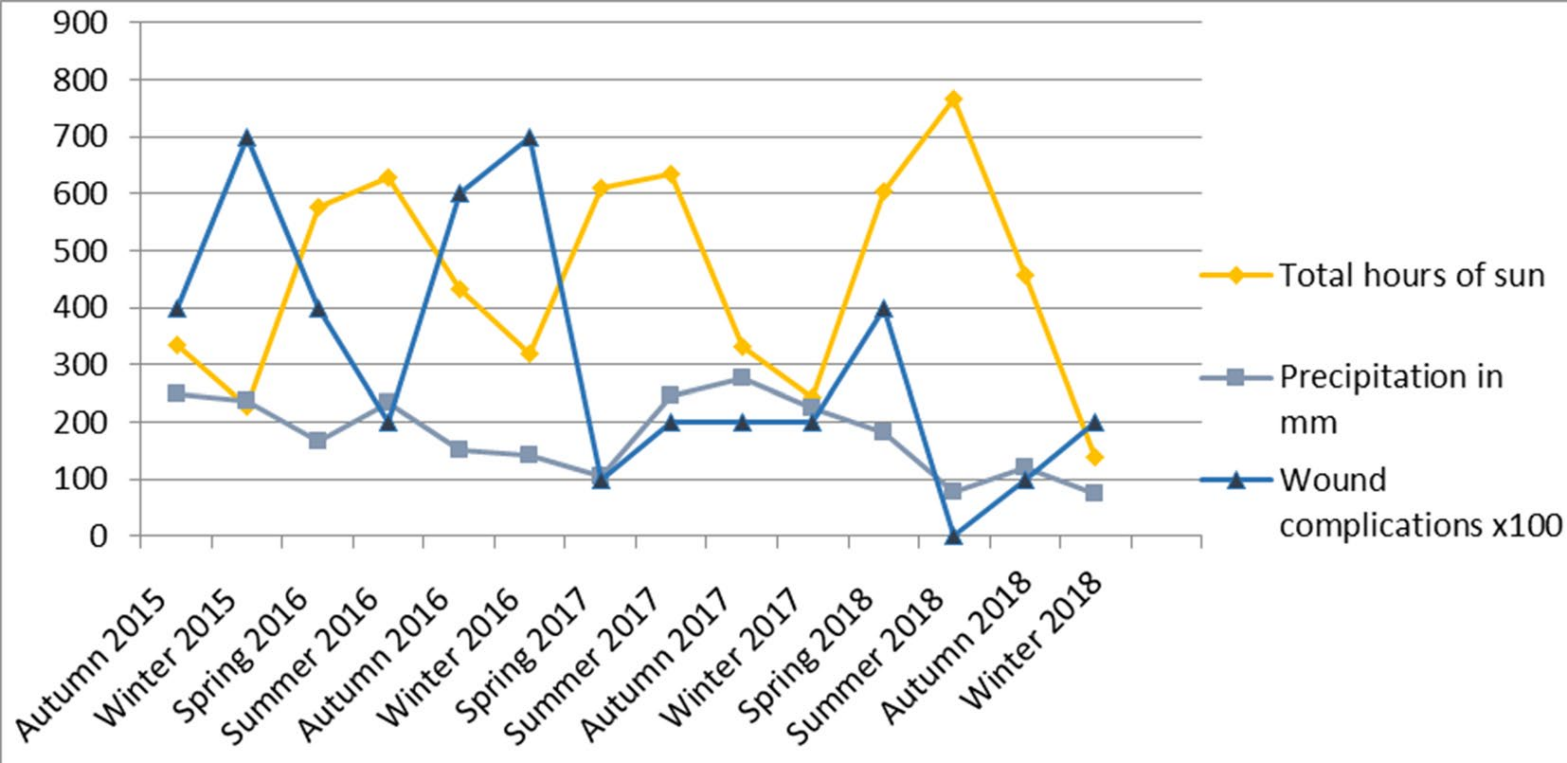
Amount of sun, precipitation and wound complications during the study period *Source* Koninklijk Nederlands Meteorologisch Instituut

Figures 2 and 3 show the average temperatures, amount of sun and precipitation of each season during the study period compared to the amount of wound complications in that time frame.

## Discussion

### Key results

The aim of this study was to identify whether the season of surgery has an influence on the incidence of wound complications following trauma surgery of the lower leg, ankle and foot. This seasonality could not be confirmed in this study, contradictory to our hypothesis. No significant difference was found in the number of surgical wound complications between seasons. Moreover, in this cohort a (non-significant) peak of wound complications was seen in winter instead of summer.

### Previous literature

To the best of our knowledge, there is only one other study which investigated seasonal effects in foot/ankle surgery. They retrospectively analyzed a large cohort of 17,939 patients undergoing orthopedic foot/ankle surgery and could not identify a statistically significant difference in wound complications between seasons, without any large confounding factors being present [15].

Previous literature on seasonality in orthopedic/trauma surgery has led to varying conclusions. Anthony et al. performed a large national retrospective cohort study on seasonal influence on 30 day infection rates after total knee (TKA) and total hip replacement (THA) surgery in the United States of America (USA). With over 750,000 included procedures, they concluded that the incidence of wound complications was highest in summer with an increase of SSIs of 24% in June compared to December, corrected for comorbidities and socioeconomic status [10]. Multiple authors have supported these findings in orthopedic and trauma surgery [9, 12, 14, 25]. However, there are also authors who did not find a difference in wound complication rates in orthopedic surgery [8, 20, 26]. This raises the question of whether or not the found effects of season are clinically relevant, even when they are statistically significant. Most of the mentioned studies reported on large populations, making it perhaps too easy to achieve statistical significance.

### Rational behind seasonality

Possible seasonality in wound complications has been ascribed to skin colonization associated with warm weather [21, 25]. The incidence of gram positive but especially gram negative bacteria such as *Escherichia coli* or *Klebsiella* seems to correlate well with the temperature, with a higher incidence in warmer months [27–31]. Sagi et al. compared the rate of wound complications in open fractures in seven different climate regions within the USA. With results of all seven regions combined they could not identify a difference in wound complications between seasons. For two out of seven regions a significant seasonal difference was found, with fall having the highest number of wound complications. Interestingly, the overall incidence of wound complications did vary significantly between climate regions (corrected for patient and injury characteristics) [19]. This leads to the hypothesis that a seasonal effect can only be found in certain climate regions. This theory is supported by Haws et al. who did not identify an overall difference in wound complications but did find an effect when comparing the wet-season with other seasons in tropical areas [26]. The temperate climate of the Netherlands might be an explanation for the fact that this study could not detect a seasonal difference in wound complications. Seasonality might only exist in climate zones with more extreme weather conditions.

Another possible explanation is the “July-effect”, a description for the start of the academic year when new (inexperienced) surgeons in training start. Previous studies have concluded that there is a significant difference in the amount of complications, morbidity, outcomes and mortality at the beginning of the academic year [32]. However, the same “July-effect” was also found in non-teaching hospitals, making training status less likely as a cause for the higher number of wound complications in summer [21]. In the presently studied hospital, surgeons in training start at varying time points during the year, making a “July-effect” unlikely to occur.

However, the reason for a difference in the incidence of wound complications between seasons is most likely multifactorial. Besides changes in temperature and bacterial flora of the skin, diet change, clothing, sunlight exposure (influence vit D or skin flora), exercise, amount of time spent inside with other people, duration and quality of sleep, exposure to diseases could also influence wound complications. Also the number of admissions of trauma surgery may vary due to weather influence such as temperature or precipitation [33].

### Limitations

Some limitations can be mentioned in this study. The first limitation is the retrospective study design, which limits the number of variables that can be reliably extracted from electronic patient records. This may have led to underreporting of confounding factors (such as “amount of blood loss”) but also of the primary outcome measure “wound complications”. Especially superficial SSIs or other minor wound healing problems may have been treated by a general practitioner and, therefore, not mentioned in patient’s hospital records. However, major wound complications needing re-admission or additional surgery were well documented. Another limitation is the heterogeneity of the study population. Although included patients have been limited to patients undergoing trauma-related surgery of foot, ankle or lower leg, there is still variation in the risk of wound complications between these locations of injury. Moreover, this study includes both elective and acute procedures and open as well as closed fractures. By reporting all potential risk factors and confounders of wound complications and incorporating them in the multivariate analysis we have tried to make it more transparent and limit the effect on the main research question.

This study may be subjected to a selection bias since it was carried out with data of a single center, specialized in complex foot/ankle traumas. This may have led to a higher overall complication percentage than one would expect. A single center study in a small country does have the advantage of similar weather conditions for each patient. Finally, due to the single center study design, the number of included patients might not be sufficient to achieve statistical significance. However, the odds that our findings with more wound complications in winter than in summer are purely coincidental and that with sufficient power we would have found an opposite effect (more wound complications in summer) are slim.

## Conclusion

Despite limitations, this study was able to confirm previous evidence and demonstrated that there is no seasonal influence on the number of wound infections in trauma-related foot/ankle surgery. Moreover, this effect of the season remained absent after correcting for multiple patient and surgery-related factors. As seasonality is multifactorial, it would be more beneficial to identify specific climate/season related factors that are of influence. Future research should focus more on these specific “sub-factors” which may be influenced (e.g. diet, sunlight/high-temperature exposure, exercise) to hopefully reduce the number of surgical wound complications. Besides season, weather conditions in the days after surgery, should, therefore, also be reported in studies on this topic.

## Appendix

See Table 5.

**Table 5:** Baseline characteristics of included and excluded patients

Variable	Included (*n* = 599)	Excluded (*n* = 167)	Statistical significance
Gender, no. (%) male	364 (60.8%)	98 (58.7%)	0.691^g^
Age, median [IQR]	46 [31–57]	45 [24–58]	0.083^h^
BMI, median [IQR]^a^	24.8 [22.7–28.1]	25.3 [22.8–30.1]	0.157^h^
ASA classification^b^			
I–II	558 (94.1%)	142 (90.3%)	0.147^g^
III–IV	35 (5.9%)	15 (9.7%)	
Diabetes, no. (%)^c^	17 (2.8%)	13 (8.0%)	0.006^g^
Immunocompromised, no. (%)^c^	13 (2.2%)	3 (1.8%)	1.000^g^
Smoking, no. (%) of smokers^d^	186 (38.8%)	32 (25.4%)	0.007^g^
Alcohol abuse (> 2 units/day), no. (%)^e^	43 (10.0%)	13 (11.2%)	0.829^g^
Drug use, no. (%)^f^	43 (10.0%)	4 (3.5%)	0.046^g^

^a^Missing 171 (124 in included, 47 in excluded)

^b^Missing 16 (6 in included, 10 in excluded)

^c^Missing 4 (in excluded)

^d^Missing 161 (120 in included, 41 in excluded)

^e^Missing 219 (168 in included, 51 in excluded)

^f^Missing 224 (170 in included, 54 in excluded)

^g^Compared with Chi-Square test

^h^Compared with Mann–Whitney *U* test

## References

[1] Modha MRK, Morriss-Roberts C, Smither M (2018). Antibiotic prophylaxis in foot and ankle surgery: a systematic review of the literature. J Foot Ankle Res.

[2] Backes M, Spierings KE, Dingemans SA (2017). Evaluation and quantification of geographical differences in wound complication rates following the extended lateral approach in displaced intra-articular calcaneal fractures: a systematic review of the literature. Injury.

[3] Owens PL, Barrett ML, Raetzman S (2014). Surgical site infections following ambulatory surgery procedures. JAMA.

[4] Metsemakers WJ, Morgenstern M, McNally MA (2018). Fracture-related infection: a consensus on definition from an international expert group. Injury.

[5] Kirkland KB, Briggs JP, Trivette SL (1999). The impact of surgical-site infections in the 1990s: attributable mortality, excess length of hospitalization, and extra costs. Infect Control Hosp Epidemiol.

[6] van Walraven C, Musselman R (2013). The surgical site infection risk score (SSIRS): a model to predict the risk of surgical site infections. PLoS ONE.

[7] WHO (2011). Report on the burden of endemic health care-associated infection worldwide.

[8] Schröder C, Schwab F, Behnke M (2015). Epidemiology of healthcare associated infections in Germany: nearly 20 years of surveillance. Int J Med Microbiol.

[9] Gruskay J, Smith J, Kepler CK (2013). The seasonality of postoperative infection in spine surgery. J Neurosurg Spine.

[10] Anthony CA, Peterson RA, Sewell DK (2018). The Seasonal variability of surgical site infections in knee and hip arthroplasty. J Arthroplasty.

[11] Rosenbaum D, Lübke B, Bauer G, Claes L (1995). Long-term effects of hindfoot fractures evaluated by means of plantar pressure analyses. Clin Biomech.

[12] Kane P, Chen C, Post Z (2014). Seasonality of infection rates after total joint arthroplasty. Orthopedics.

[13] Durkin MJ, Dicks KV, Baker AW (2015). Seasonal variation of common surgical site infections: does season matter?. Infect Control Hosp Epidemiol.

[14] Koljonen V, Tukiainen E, Pipping D, Kolho E (2009). “Dog days” surgical site infections in a Finnish trauma hospital during 2002–2005. J Hosp Infect.

[15] Huntley SR, Lee S, Kalra R (2018). Associations between season and surgical site infections in orthopaedic foot and ankle surgery. Foot.

[16] Sagi HC, Cooper S, Donahue D (2015). Seasonal variations in posttraumatic wound infections after open extremity fractures. J Trauma Acute Care Surg.

[17] Ng M, Song S, George J (2017). Associations between seasonal variation and post-operative complications after total hip arthroplasty. Ann Transl Med.

[18] Parkinson B, Armit D, Mcewen P (2018). Is climate associated with revision for prosthetic joint infection after primary TKA?. Clin Orthop Relat Res.

[19] Sagi HC, Donohue D, Cooper S (2017). Institutional and seasonal variations in the incidence and causative organisms for posttraumatic infection following open fractures. J Orthop Trauma.

[20] Malik AT, Azmat SK, Ali A (2018). Seasonal influence on postoperative complications after total knee arthroplasty. Knee Surg Relat Res.

[21] Anthony CA, Peterson RA, Polgreen LA (2017). The seasonal variability in surgical site infections and the association with warmer weather: a population-based investigation. Infect Control Hosp Epidemiol.

[22] Taplin D, Zaias NRG (1965). Environmental influences on the microbiology of the skin. Arch Environ Health.

[23] Berrios-Torres S, Umscheid C, Bratzler D (2017). Centers for disease control and prevention guideline for the prevention of surgical site infection, 2017. JAMA Surg.

[24] IBM Corp Released (2017). IBM SPSS statistics for windows, version 25.0.

[25] Durkin MJ, Dicks KV, Baker AW (2015). Postoperative infection in spine surgery: does the month matter?. J Neurosurg Spine.

[26] Haws BE, Braun BM, Creech TB (2017). Is there a seasonal influence on orthopaedic surgical wound infection rates?. J Surg Orthop Adv.

[27] Wang X, Towers S, Panchanathan S, Chowell G (2013). A population based study of seasonality of skin and soft tissue infections: implications for the spread of CA-MRSA. PLoS ONE.

[28] Leekha S, Diekema DJ, Perencevich EN (2012). Seasonality of staphylococcal infections. Clin Microbiol Infect.

[29] Anderson DJ, Richet H, Chen LF (2008). Seasonal variation in *Klebsiella pneumoniae* bloodstream infection on 4 continents. J Infect Dis.

[30] Perencevich EN, McGregor JC, Shardell M (2008). Summer peaks in the incidences of gram-negative bacterial infection among hospitalized patients. Infect Control Hosp Epidemiol.

[31] Freeman JT, Anderson DJ, Sexton DJ (2009). Seasonal peaks in *Escherichia coli *infections: possible explanations and implications. Clin Microbiol Infect.

[32] Englesbe MJ, Pelletier SJ, Magee JC (2007). Seasonal variation in surgical outcomes as measured by the American College of Surgeons-National Surgical Quality Improvement Program (ACS-NSQIP). Ann Surg.

[33] Bhattacharyya T, Millham FH (2001). Relationship between weather and seasonal factors and trauma admission volume at a level I trauma center. J Trauma Inj Infect Crit Care.

